# Disparate Time-to-Treatment and Varied Incidence of Actionable Non-Small Cell Lung Cancer Molecular Alterations in British Columbia: A Historical Cohort Study

**DOI:** 10.3390/curroncol30010012

**Published:** 2022-12-22

**Authors:** Roy Avraham Hilzenrat, Stephen Yip, Barbara Melosky, Cheryl Ho, Janessa Laskin, Sophie Sun, James J. Choi, Anna L. McGuire

**Affiliations:** 1 Department of Surgery, Division of Thoracic Surgery, Vancouver Coastal Health, University of British Columbia, 2775 Laurel Street, Vancouver, BC V5Z 1M9, Canada; 2 Cancer Genetics & Genomic Laboratory, BC Cancer—Vancouver Centre, 600 West 10th Avenue, Vancouver, BC V5Z 4E6, Canada; 3 Department of Medicine, Division of Medical Oncology, BC Cancer Vancouver Centre, University of British Columbia, 600 West 10th Avenue, Vancouver, BC V5Z 4E6, Canada; 4 Vancouver Coastal Health Research Institute, 2635 Laurel Street, Vancouver, BC V5Z 1M9, Canada

**Keywords:** non-small cell lung cancer, thoracic malignancy, cancer care, driver mutations, British Columbia

## Abstract

Background: non-small cell lung cancer (NSCLC) outcomes remain suboptimal for early-stage disease despite emerging advances in systemic therapy for the peri-operative period. Next-generation sequencing (NGS) identifies driver mutations for which targeted therapies have been developed that improve survival. The BC lung cancer screening program, which was initiated in May 2022, is expected to identify people with early and late stages of NSCLC. It is crucial to first understand the molecular epidemiology and patterns of time to initiate treatment across its five health authorities (HA) to optimize the delivery of care for NSCLC in BC. In this way, we may harness the benefits of targeted therapy for more people with NSCLC as novel advances in therapy continue to emerge. Objective: to compare (a) the frequency of actionable NSCLC molecular alterations among HAs and (b) the time to treatment initiation. Methods: a retrospective observational study was conducted with prospectively collected data from the BC CGL Database. Adults with late stage NSCLC who underwent targeted NGS were included for the time period from May 2020 to June 2021. Demographics, actionable molecular alterations, PDL-1 expression, and time to treatment across HAs were examined. Using appropriate statistical tests for comparison among HAs, p>0.05 was deemed significant. Results: 582 patients underwent NGS/IHC and analysis during the study period. The mean age was 71 (10.1), and 326 (56%) patients were female. A significantly higher proportion of all EGFRm+ were identified within Vancouver Coastal Health (VCHA) and Fraser Health Authority (FHA) compared to the other health authorities (*p* < 0.001). This also holds true for common sensitizing EGFRm+ alone (*p* < 0.001) and for sensitizing EGFRm+ when adjusted for females and smoker status (OR 0.75; 95% CI 0.62, 0.92; *p* = 0.005). Patients residing within the Northern, Interior, and Island HAs were less likely to receive treatment at the same rate as those in VCHA and FHA HAs. Conclusion: actionable NSCLC driver mutations are present in all regional HAs, with disparity noted in time to initiate treatment between HAs. This provides evidence for the importance of molecular testing for patients in all BC HAs to guide personalized and timely NSCLC treatment.

## 1. Introduction

### 1.1. Background

Lung cancer is the most common malignancy in Canada that is responsible for more cancer deaths among Canadians than colorectal, breast, and prostate cancer combined [1]. It is responsible for 24.3% of all cancer deaths in Canada, and is expected to remain the leading cause of cancer death for both males and females [1]. The World Health Organization (WHO) classifies lung cancer into two broad categories based on tumor biology, treatment, and prognosis: (1) non-small-cell lung cancer (NSCLC) and (2) small-cell lung cancer (SCLC). Early diagnosis and treatment are important to optimize patient survival as only those with early-stage NSCLC (stage I/II/III) are amenable to curative intent treatment. Regarding early detection, the initiation of lung cancer screening programs in Canada and internationally are expected to identify individuals with both early and late stage NSCLC [2]. Ongoing advances in perioperative targeted systemic therapy are also crucial as up to 55% of NSCLC patients develop recurrent metastatic disease following early-stage tumor resection with their survival decreased as a result. The majority of tumors have been shown to express a molecular alteration [3]. Emerging targeted therapies for driver mutations and immunotherapy, in particular, are changing the landscape of treatment options in early stage neoadjuvant and adjuvant space for NSCLC [4,5,6,7].

Adjuvant use of the third generation EGFR Tyrosine Kinase Inhibitor Osimertinib has recently been shown to improve overall survival in common epidermal growth factor receptor (EGFR) mutation NSCLC [4]. Promising results have supported its evaluation in the neoadjuvant setting (ClinicalTrials.gov Identifier: NCT04351555). Adjuvant Nivolumab immunotherapy has also recently shown substantially improved disease-free survival compared to standard adjuvant chemotherapy [5,6]. The rapidly evolving landscape of multimodal NSCLC management highlights the importance of investigating the molecular epidemiology of NSCLC patients. Next-generation-sequencing (NGS) of early-stage NSCLC for genetic alterations following surgical resection is not currently done in many Canadian provinces or institutions internationally, despite the demonstrated survival benefit with Osimertinib for common sensitizing tyrosine kinase inhibitors to EGFR [8]. In contrast, NSCLC patients with late stage metastatic or recurrent disease following pulmonary resection are eligible for timely molecular testing to guide personalized, systemic therapy choices for clinically actionable genetic alterations. Such actionable alterations include EGFR, anaplastic lymphoma kinase (ALK), met proto-oncogene (MET), and c-ROS proto-oncogene 1 (ROS1) [9,10,11,12,13,14,15,16,17,18,19]. Additional novel targeted therapeutics are in development, such as those for kirsten rat sarcoma viral oncogene homolog G12C (KRAS G12C) [14,19,20].

Routine testing for EGFR mutations and ALK rearrangements has become a standard of care for advanced non-squamous NSCLC [21]. However, jurisdictional implementation of reflexive molecular testing remains an unsolved challenge. Only 38% of eligible NSCLC patients in a 2010 Canadian EGFR testing program underwent molecular testing [22]. A large Canadian university hospital, where EGFR testing for all advanced non-squamous NSCLC had been implemented in 2006, reported a 342-day median time to the detection of EGFR mutation after reviewing its program from its initiation to 2019 [23]. Canadian lung cancer experts have developed strategies to mitigate challenges in molecular testing to promote the best possible and most personalized care [21,24,25]. Local healthcare systems must promote these recommendations and strategies regarding reflexive molecular testing through local epidemiologic studies to characterize targetable NSCLC molecular alterations. Toronto’s University Health Network has rightfully studied their rates of targetable NSCLC mutations [26], reporting a KRAS and EGFR mutation frequency of 32.3% and 24.2%, respectively. Yet there remains a paucity of local Canadian NSCLC driver mutation epidemiological studies to guide targeted treatment efforts.

The BC lung cancer screening program, initiated in May 2022, is expected to identify people with early and late stages of NSCLC. Delivery of NSCLC care in BC is regionalized across five health authorities (HAs), with a centralized NGS at the BC Cancer Genomic Lab (CGL). It is crucial to first understand the molecular epidemiology and patterns of time to treatment across HAs to optimize the delivery of care for NSCLC in BC and respond to national NSCLC molecular testing optimizing strategies. In this way, we may harness the benefits of targeted therapy for more people with NSCLC as novel advances in therapy continue to emerge.

In conjunction with the advances seen within the realm of NSCLC targeted therapy and the initiation of the BC cancer lung cancer screening program in May 2022, we aim to deepen our understanding of the molecular epidemiology of common actionable NSCLC genetic alterations within the BC health region. The delivery of NSCLC care in BC is regionalized geographically with five health authorities (HAs): Vancouver Coastal Health Authority (VCHA), Fraser Health Authority (FHA), Interior Health Authority (IHA), Northern Health Authority (NHA), and the Island Health Authority (IsHA). NGS testing and reporting is centralized at the BC Cancer Genomic Lab (CGL) for all five HAs. It is crucial to first understand the molecular epidemiology and patterns of time to treatment across HAs to optimize the delivery of care for NSCLC in BC.

### 1.2. Objective

The study objective is two-fold. It seeks to make comparisons among BC’s regional HAs, including (a) the frequency of actionable NSCLC molecular alterations and (b) the time to treatment initiation. In this way, we may harness the benefits of targeted therapy for more people with NSCLC as novel advances in therapy continue to emerge and more eligible patients are identified through lung cancer screening.

## 2. Methods

### 2.1. Study Design and Setting

A retrospective cohort study was conducted at BC Cancer Vancouver Centre . Tumor molecular data and clinical variables were collected from May 2020 to June 2021. This study, #H18-03295-A009, was approved by the BC Cancer Institutional Research Ethics Board.

### 2.2. Participants and Data Sources

A retrospective analysis was conducted using prospectively collected demographic and tumor molecular data for NSCLC patients from the BC Cancer Genomic Lab dataset whose tumor tissue was subject to NGS during the 12-month study period (18 May 2020–1 June 2021). All patients had late stage or recurrent NSCLC (based on the American Joint Commission on Cancer [AJCC] Staging 8th Edition) [27], and as such, they were eligible for molecular testing through the BC Cancer CGL via the standard of care. Patients with squamous NSCLC were eligible for NGS only if they were never-smokers. Additional variables retrieved retrospectively from the medical record included: smoking status (never, former, or current) and immunohistochemistry for PD-L1 (reported as <1%, 1–49%, and >50%).

### 2.3. Primary Outcomes

Patients with clinically actionable NSCLC genetic alterations were identified. Actionable driver mutations were defined as those with targeted therapies available in North America. EGFR mutations were interpreted as common sensitizing mutations (EGFR Exon 19 deletion and EGFR L858R), uncommon sensitizing mutations (E709X, G719X, S768I, L861Q, and EGFR co-mutations of the latter), and uncommon non-sensitizing mutations (exon 20 insertion, Denovo T970M). Health authority was determined geographically with tabulation of the participants’ residential postal codes. Time to treatment was defined as the time from the date of the diagnostic procedure to the date of first NSCLC treatment.

### 2.4. Targeted NGS Panels for NSCLC Genetic Alterations

The DNA-based hybrid-capture multiplex NGS assay (“OncoPanel”,BC Cancer Vancouver Centre) from the Cancer Genetics and Genomic Laboratory (CGL) at BC Cancer Vancouver Centre was utilized for the cohort. Details regarding the CGK OncoPanel and gene targets are available at http://cancergeneticslab.ca/genes/oncopanel/ and in the supplementary data section. In brief, genomic DNA was extracted using an automated system (Promega Maxwell) followed by FFPE repair, ligation-based library construction, PCR amplification, hybridization capture, and sequencing on a HiSeq2500 platform (Illumina, San Diego, USA). Single-strand consensus sequences were generated from UMI-indexed reads using fgbio and aligned with the GRCh37 human genome reference using BWA. Variant calling of DNA mutations and insertions/deletions (INDELs) was performed using samtools and VarScan2. Annotation and filtering of variants was performed using Agilent’s Alissa Interpret platform. For gene fusions, immunohistochemistry (IHC) was employed to determine the aberrant protein expression of ALK, RET, and ROS1 status from matched FFPE slides.

### 2.5. Statistical Methods

Continuous variables were summarized using means and standard deviations and analyzed using the Student’s *t*-test. Categorical variables were expressed as frequencies and percentages and compared using the Chi-square or Fisher’s exact tests if appropriate. Single-factor and multi-factor analyses in relation to outcomes (*EGFR*, for example) required the use of logistic regression. Variables identified to include in the model based on the literature search included female sex and smoking status as these have known associations with sensitizing EGFRm+ expression^18^. Missing data were not imputed, and out of province records were treated as missing data.

A Cox-Proportional Hazard analysis was performed to assess the differences in time-to-treatment among the health authorities. The model was adjusted for age, sex, type of treatment, and stage at presentation. The conventional level of statistical significance (*p* < 0.05) was used throughout the study as an indicator of a potential effect. All tests were two-sided. Statistical analyses were performed with Stata17 (StataCorp. 2021. Stata Statistical Software: Release 17. College Station, TX: StataCorp LLC.) and R (R Core Team 2020, R: A language and environment for statistical computing. R Foundation for Statistical Computing, Vienna, Austria).

## 3. Results

**Figure 1:**
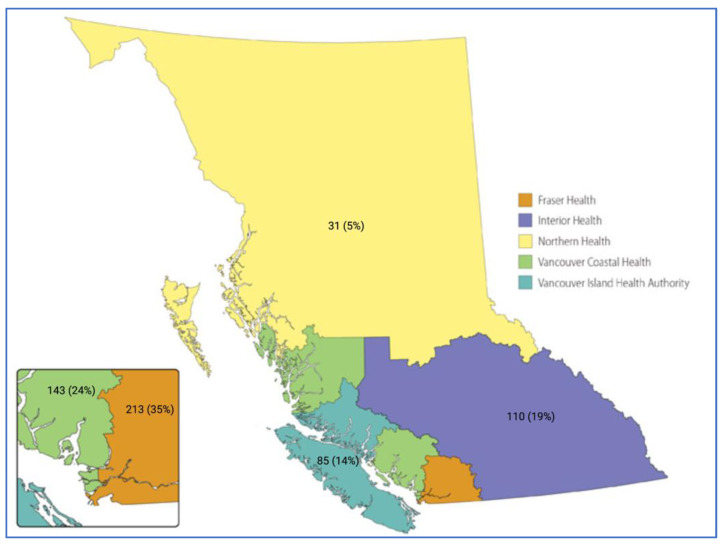
Depicted is a rendering of the province of British Columbia (www2.gov.bc.ca, accessed on 17 December 2022). Each BC provincial health authority is color labeled as indicated in the legend, and n (%) for each respective health authority depicts the proportion of late stage NSCLC patients who underwent molecular profiling at the BC Cancer Genomic Lab during the study period (May 2020 to June 2021). The 11 (1.9%) out of province records are not depicted.

A total of 593 NSCLC patients met eligibility criteria, of which 333 (56.2%) were female, with a median age of 72 (65, 78). VCHA (143, 24.1%) and FHA (213, 35.9%) harbored the majority of study participants as depicted in Figure 1. There were 11 (1.9%) out of province records excluded from the comparative analysis. A molecular alteration was detected in tumor tissue in 540 (92.8%) of the 582 patients who underwent analysis. A clinically actionable moleculat alteration was identified in 264 (45.4%) of the patients.

Adenocarcinoma was the most common NSCLC subtype (482, 82.8%). Of the 582 study participants, 257 (44.2%) were found to have clinically actionable molecular alterations with testing, and 185 (32%) presented with late stage metastatic disease. Demographics and NSCLC tumor characteristics are summarized in Table 1. There was a significantly higher proportion of never and former smokers in the VCHA and FHA (*p* < 0.001) as well as adenocarcinoma histology (*p* = 0.03). All other characteristics noted were reasonably balanced between HAs, including PD-L1.

A summary of the incidence of actionable molecular alterations identified using oncopanel and IHC during the study period are summarized in Table 2. Any *EGFR* mutation was detected in 105 patients, and KRAS G12C mutations were identified in 108 (18.6%) individuals. A patient’s tumor expressed multiple mutations in several instances; in particular, co-mutations were observed in the uncommon sensitizing EGFR mutations. EGFR and KRAS mutations were mutually exclusive. ALK fusions detected by IHC were noted to co-occur with KRAS mutations in our cohort.

Univariate analyses showed that there was a significantly higher proportion of common sensitizing EGFR mutations (EGFR exon 19 deletion and EGFR L858R, for example) identified in VCHA and FHA compared to other health authorities (*p* < 0.001). There was no appreciable difference in the proportion of uncommon sensitizing and uncommon non-sensitizing EGFR mutations among the HAs. As anticipated, counts for these uncommon EGFR mutations in the cohort were low overall. A persistent strong association with these HAs remained when the multivariable analysis adjusted for the association of sensitizing EGFR mutations with the female sex (OR 0.65; 95% CI 0.54, 0.80; *p* < 0.001). However, after adjusting for never smoker status, the observed association between the higher proportion of sensitizing EGFR mutations in VCHA and FHA was weakened (OR 0.75; 95% CI 0.62, 0.92; *p* = 0.005).

There was an overall difference in KRAS mutation incidences observed between health authorities on univariable analysis. There was no significant difference observed following multivariable analysis and adjusting for smoker status on KRAS incidence by health authority (OR 1.1; 95% CI 0.97, 1.3; *p* = 0.14).

There were otherwise no appreciable differences in incidences of MET exon 14 skip, ERRB2 (HER2), and BRAF V600E mutations or fusion profiles among HAs, although counts for these mutations in the study cohort were low.

The Cox Proportional Hazard analysis suggests that NSCLC patients residing in regions within IHA (HR 0.60; 95% CI 0.38, 0.95), NHA (HR 0.45; 95% CI 0.21–0.96), and isHA (HR 0.57; 95% CI 0.35, 0.93), experience greater treatment wait times than that of VCHA and FHA when adjusting for sex, age, treatment type, and stage at presentation. There was no significant difference between wait times in VCHA and FHA (HR 0.77; 95% CI 0.52,1.12).

## 4. Discussion

NSCLC mutations govern the disease’s associated survival and response to treatment, including mutation-targeted and immune-therapy [28]. Apart from its subtype, NSCLC’s driving mutation(s) are argued to be the most clinically significant disease characteristic. This is particularly true for EGFR mutations given recent advances in the development of its targeted therapies. The favourable outcomes reported for both late stage and early stage cancer from tyrosine kinase inhibitors (TKIs) targeted for EGFR mutated NSCLC highlight the importance of summarizing the frequency of such mutations among populations [4]. Although an individual patients’ journey with targeted therapy may begin with an oncology specialist referral to the Cancer Genomics and Genetics labs, knowledge translation and resource allocation is managed by regional health authority systems which rely on population data and mapping. To our knowledge, this is the first study to describe the molecular epidemiology of clinically actionable molecular alterations with HA subgroup analyses for the province of BC. In May 2022, BC Cancer launched Canada’s first province-wide lung cancer screening program, providing access to eligible individuals at 36 provincial sites [29]. Typically, a higher rate of early-stage disease is often captured when screening programs are initiated, which allows for curative intent therapy. In the context of the promising results of adjuvant and neoadjuvant TKIs use for improving overall and disease-free survival, early detection of lung cancer is likely to provide additional survival benefits in the contemporary management of lung cancer. This study provides a mapping of EGFR mutation frequencies among BC’s health authorities, and it may subsequently aid to allot physician education and patient resources accordingly to maximize the quality of provincial NSCLC care.

This retrospective review of NSCLC genomics data from the BC Cancer Genomic Lab Oncopanel Dataset summarizes the frequency of actionable driver mutations (EGFR, KRAS G12C, MET Exon 14 skip, ALK fusion, and ROS1 fusion), PDL-1 expression, and treatment wait times among five health authorities. EGFR mutations were present in 106 (17.9%) participants, which is similar to proportions described in contemporary literature. The incidence of EGFR mutations in NSCLC has been shown to vary among ethnicities, occurring at a rate of 15–20% in North Americans [25,30,31], 5–12% in Europeans [32,33], 19% in African Americans [34], and 26–51% among Asian populations, such as those with Chinese, Korean, or Japanese backgrounds [35,36,37]. Common EGFR mutations, defined as exon 19 deletion and exon 21 codon 858 point mutation (L858R), were present in 80.8% of the patients with EGFR mutations, which aligns with rates described among other groups [32,38]. Significantly higher proportions of all EGFR mutations were identified in VCHA and FHA compared to other health authorities, which may be driven by differences in never smoker status population demographics across the province. Asian ethnicity is associated with a greater likelihood of having EGFR mutations NSCLC [39]; hence, regions with increased Asian population densities are expected to harbor greater proportions of EGFR mutations. As per Canada’s most recent Census Profile (2016) [40], 86% of British Columbia’s Asian population lives in the Vancouver census metropolitan area, legitimizing the observed difference in EGFR mutation frequencies among health authorities.

Another notable observation in this study is the limited number of patients represented from BC’s Northern Health Authority. As noted above, the BC Cancer CGL dataset for the study period represents lung cancer patients who were referred for centralized NGS molecular testing following a diagnosis of metastatic or recurrent lung cancer. The small number of NHA NSCLC patients available in the dataset (31, 5%) raises the question of whether NSCLC molecular testing referral patterns are similar across health authorities and whether certain regions face disproportionate referral challenges. Despite accounting for the smaller population in NHA, its representation in the dataset is still less than expected based on regional population matching [41]. While the scope of this study does not specifically address issues relating to referral pathways, it highlights the necessity to investigate potential disparities between health authorities as it may have implications for NSCLC patients harboring actionable driver mutations who would otherwise be eligible for life-extending therapies.

Local health authorities are also responsible for appropriate treatment wait times and must allocate resources to ensure equitable cancer care throughout the province. Delayed access to therapy following diagnosis, adjusting for stage at presentation, is associated with worse overall survival [42]. The timeliness of care for patients with lung cancer has been addressed by cancer societies across the globe. The acceptable time interval of 30–52 days from diagnosis to first treatment has been acknowledged by the British Thoracic Society [43], UK National Health Service [44,45,46], Rand Corporation [47,48], American College of Chest Physicians [49], and Cancer Care Ontario [50]. The median time to treatment, defined as the time from diagnostic procedure (CT-guided biopsy, endobronchial biopsy, pleural fluid cytology, or imaging) to first treatment (surgery, oral targeted therapy, radiation, or chemotherapy) in this study was 39 days (IQR 27, 63) across BC (Table 3). A British Columbian retrospective study by Van de Vosse et al. reviewed wait times of lung cancer patients from southern IHA in 2010–2011. They reported a median time from biopsy to first treatment of 26 days [51] which varies from our observed median wait time of 55 days in IHA. This difference can be explained by the geographic limitations of Van de Vosse’s study population as they only included patients from a sub-region of IHA who are closer to major thoracic oncology centers south of the region, such as Kelowna General Hospital and its cancer agency.

The COX proportional hazard time-to-event analysis revealed increased wait times for NSCLC patients residing outside of FHA and VCHA regions. This may be explained by the population density of these regions and their proximity to centralized oncology care, including thoracic surgical services. NSCLC patients residing in IHA and NHA experienced the greatest wait times, with median wait times of 55 and 64 days, respectively. This study was not designed to elicit potential causes of differences in wait times, yet this is an important finding that warrants further investigation. Diagnostic and therapeutic timelines are lengthy, multidisciplinary, and complex, which creates many points at which care may be delayed. Therefore, system-wide changes are often required to address suboptimal wait times. The introduction of allied health programs, including lung cancer “nurse navigators” and the development of lung nodule rapid assessment programs, have been demonstrated to reduce surgical and medical treatment wait times for lung cancer patients [52]. HAs may consider introducing care navigators to support patients and clinicians through the lung cancer diagnostic work-up and treatment journey.

This study is not without limitations. The analysis is retrospective in nature which subjects it to inherit bias, despite the prospective nature of molecular data acquisition. The sample size was limited by the number of patients referred for molecular testing at the CGL. A greater number of NSCLC patients will have data regarding their cancer’s genomic details as EGFR TKIs become more widely accepted for adjuvant and potentially neoadjuvant therapy, which will also enable greater sample sizes and confidence in results. Regarding the adjusted time-to-treatment analysis, certain variables could not be captured or controlled for, including system factors and delays in treatment due to patient hesitation or indecision. Furthermore, a proportion of the study cohort data was collected during the early stages of the COVID-19 pandemic which may have impacted patient treatment choice and potential delays in treatment.

## 5. Conclusions

We reported significant differences in the molecular epidemiology of TKI sensitizing EGFR mutated NSCLC in British Columbia. The observed frequency was significantly higher in VCHA and FHA compared to other HAs, as was the proportion of never smokers in the population. Further investigation will be valuable to clarify reasons for the noted differences in molecular testing referral patterns across HAs and system workflow disparities that may account for observed differences in time to treatment between regions for patients with lung cancer.

## Figures and Tables

**Table 1:** Demographic and tumor characteristics for the total cohort (May 2020 to June 2021) and actionable NSCLC gene alterations.

Characteristic	Total (n = 582)	VCHA	FHA	IHA	NHA	isHA	*p*-Value *
		(n = 143)	(n = 213)	(n = 110)	(n = 31)	(n = 85)	
**Female, n (%)**	326 (56)	87 (60.8)	107 (50.2)	70 (63.6)	17 (54.8)	45 (52.9)	0.19
**Male, n (%)**	256 (44)	56 (39.2)	106 (49.8)	40 (36.4)	14 (45.2)	40 (47.1)	
**Age at presentation, mean (SD)**	71 (10.1)	70 (11.7)	72 (10.3)	71 (9.0)	72 (8.2)	72 (8.7)	0.29 **
**Smoking status, n (%)**							<0.001
Never smoker	121 (20.4)	49 (34.3)	45 (21.1)	11 (10.0)	2 (6.5)	14 (16.5)	
Former smoker	351 (59.2)	71 (49.6)	130 (61.0)	73 (66.4)	19 (61.3)	54 (63.5)	
Current smoker	104 (17.5)	21 (14.7)	37 (17.4)	20 (18.2)	10 (32.3)	14 (16.5)	
No record	17 (2.9)	2 (1.4)	2 (1.4)	6 (5.5)	-	3 (3.5)	
**NSCLC histology, n (%)**							0.03
Adenocarcinoma	482 (82.8)	119 (83.2)	176 (82.6)	87 (79.1)	28 (90.3)	72 (84.7)	
Squamous cell	10 (1.7)	5 (3.5)	3 (1.41)	-	-	2 (2.4)	
Adenosquamous carcinoma	4 (<1)	1 (0.7)	2 (0.9)	1 (0.9)	-	-	
NOS	81 (13.9)	18 (12.6)	32 (15.0)	21 (19.1)	3 (9.7)	7 (8.2)	
Large cell carcinoma	2 (<1)	-	-	1 (0.9)	-	1 (1.2)	
Sarcomatous carcinoma	3 (<1)	-	-	-	-	3 (3.5)	
**Any molecular alteration detected, n (%)**	540 (92.8)	134 (93.7)	197 (92.5)	104 (94.6)	26 (83.9)	79 (93)	0.36
**Actionable NSCLC molecular alteration, n (%)**	264 (45.4)	73 (51.1)	100 (47)	42 (38.2)	17 54.8)	35 (41.2)	0.29
**PDL1 expression, n (%)**							0.49 **
<1%	212 (36.4)	46 (32.2)	92 (43.2)	36 (32.7)	12 (38.7)	26 (30.6)	
1–49%	128 (22.0)	34 (23.8)	43 (20.2)	23 (20.9)	6 (19.4)	22 (25.9)	
>50%	233 (40.0)	59 (41.2)	75 (35.2)	49 (44.6)	13 (41.9)	37 (43.5)	
Not performed/no record	9 (1.6)	4 (2.8)	3 (1.4)	2 (1.8)	-	-	

NSCLC, non-small cell lung cancer; NOS, not otherwise specified; VCHA, Vancouver Coastal Health Authority; FHA, Fraser Health Authority; IHA, Interior Health Authority; NHA, Northern Health Authority; isHA, Island Health Authority; PDL1 = programmed death-ligand 1. The 11 out of province individuals have been excluded from analysis. * Chi-square or Fisher’s exact test; ** t-test.

**Table 2:** Incidences of actionable molecular alterations for the total BC Cancer NSCLC cohort (May 2020 to June 2021) and health authority subgroups.

	Total (n = 582)	VCHA	FHA	IHA	NHA	isHA	*p*-Value *
		(n = 143)	(n = 213)	(n = 110)	(n = 31)	(n = 85)	
**Patient with at least one actionable NSCLC**	264 (45.4)	73 (51.1)	100 (47)	42 (38.2)	17 (54.8)	35 (41.2)	0.29
**molecular alteration**							
**n (%)**							
**Any EGFRm +**	105 (18%) EGFRm+ in 582	42 (70.6)	42 (19.7)	11 (10)	-	10 (11.8)	<0.001
**n (%)**	patients						
**Description of EGFRm subtype detected overall and by a health authority (one patient may have >1 EGFRm detected), n (%)**							
Common sensitizing EGFRm+	84 (14.4) mutations	35 (24.5)	34 (16)	10 (9.1)	-	5 (5.9)	<0.001
EGFR exon 19 deletion							
EGFR L858R	41 (7)						
	43 (7.4)	19 (13.2)	17 (8)	4 (3.6)	-	1 (1.2)	
		16 (11.2)	17 (8)	6 (5.45)	-	4 (4.7)	
Uncommon sensitizing EGFRm+	16 (2.8) mutations	3 (2.1)	8 (3.8)	2 (1.8)	-	3 (3.53)	0.35
EGFR G709X	4						
EGFR G719X	9	1	1	1	-	1	
EGFR S768I	7	1	4	2	-	3	
EGFR L861Q	3	-	5	1	-	1	
EGFR co-mutation **	12 **	1	2	-	-	-	
		2 **	6 **	2 **	-	2 **	
Uncommon non-sensitizing EGFRm+	11 (1.9) mutations	6 (4.2)	3 (1.4)	-	-	2 (2.4)	0.13
EGFR exon 20 insertion							
Denovo T790M	9 (1.6)						
		6 (4.2)	2 (0.9)	-	-	1 (1.2)	
	2 (0.3)						
		-	1 (0.5)	-	-	1 (0.5)	
**Non-EGFR molecular alterations detected overall and by a health authority, n (%)**							
**Any KRASm+**	236 (40.6)	49 (34.3)	78 (36.6)	55 (50)	17 (54.8)	37 (43.5)	0.03
KRAS G12C	108 (18.6)	17 (11.9)	40 (18.8)	24 (21.8)	10 (32.3)	17 (20)	0.06
MET exon 14 skip	17 (2.9)	6 (4.2)	5 (2.4)	1 (0.91)	2 (6.5)	3 (3.5)	0.39
ERRB2 (HER2)	13 (2.2)	6 (4.2)	3 (1.4)	2 (1.8)	1 (3.2)	1 (1.2)	0.43
BRAF V600E	14 (2.4)	1 (0.7)	8 (3.8)	1 (0.9)	1 (3.2)	3 (3.5)	0.29
**Fusion +**	14 (2.4)	3 (2.1)	4 (1.9)	3 (2.73)	1 (3.2)	3 (3.5)	0.92
ALK	13 (2.2)	3 (2.1)	4 (1.9)	3 (2.73)	1 (3.2)	2.35	
ROS1	1 (0.2)	-	-	-	-	1 (1.2)	

Out of province: n = 2 excluded from the health authority subgroups; * Chi-square or Fisher’s exact test; NSCLC, non-small cell lung cancer; ** EGFR “co-mutation” of more than one of the above noted uncommon sensitizing EGFRm.

**Table 3:** Time to treatment among health authority sub-groups.

Health Authority	Time to Treatment—Median (days) [IQR]
Overall	39 (27, 63)
VCHA	40.5 (28.5, 61.75)
FHA	36 (25, 49)
IHA	55 (30, 75.5)
NHA	64 (48, 71)
isHA	36 (22, 68)

VCHA, Vancouver Coastal Health Authority; FHA, Fraser Health Authority; IHA, Interior Health Authority; NHA, Northern Health Authority; isHA, Island Health Authority.

## Data Availability

The data presented in this study are available on request from the corresponding author.

## Supplementary Materials

The supporting information can be downloaded at: https://www.mdpi.com/article/10.3390/curroncol30010012/s1. Supplementary Data: OncoPanel from the Cancer Genetics and Genomics Laboratory (CGL) at BC Cancer.

## Abbreviations

AJCC	American Joint Commission on Cancer
ALK	Anaplastic lymphoma kinase
BC	British Columbia
EGFR	Epidermal growth factor receptor
FHA	Fraser Health Authority
FDG-PET	Fluorodeoxyglucose positron emission tomography
FFPE	Formalin fixed paraffin embedded
HA	Health authority
ICR	Interquartile range
IHA	Interior Health Authority
IsHA	Island Health Authority
MET	Met proto-oncogene
NSCLC	Non-small cell lung cancer
RET	Ret proto-oncogene
ROS1	c-ROS proto- oncogene 1
SCLC	Small cell lung cancer (SCLC)
TKI	Tyrosine kinase inhibitor (TKI)
VCC	Vancouver Cancer Centre
VCHA	Vancouver Coastal Health Authority
WHO	World Health Organization
