# Supplementary material for: Disparate Time-to-Treatment and Varied Incidence of Actionable Non-Small Cell Lung Cancer Molecular Alterations in British Columbia: A Historical Cohort Study
Source: Curr Oncol. 2022 Dec 22;30(1):145–56. doi: 10.3390/curroncol30010012 (PMC9858228; doi:10.3390/curroncol30010012)
Supplement: Supplementary file 1 [file curroncol-30-00012-s001.zip › curroncol-2110642-supplementary.pdf]

Supplementary Data – OncoPanel from the Cancer Genetics and Genomics Laboratory (CGL) at BC Cancer http://cancergeneticslab.ca/genes/oncopanel/

CGL's Oncopanel is a next-generation sequencing assay that is offered to provide predictive, prognostic, and diagnostic information for patients with a variety of solid tumours. All the genes targeted by this assay (see below) are screened for DNA changes (variants). The clinical significance of variants or variant combinations is then interpreted in the context of therapy-response, prognosis, or diagnostic criteria.

Sequencing and informatics services for this assay are provided by PHSA's Centre for Clinical Genomics (CCG) and results are interpreted by CGL. Single-base and small insertion/deletion variants are screened in the targeted gene regions and variant classification is adapted from AMP/ASCO/CAP guidelines (Li (2017) PMID:27993330). Oncopanel does not detect gene fusions or copy-number variants.

Indications for Lung Cancer OncoPanel in BC
Solid tumor testing is available for Stage IIIB/IV non-small cell, non-neuroendocrine lung adenocarcinoma, for patients both prior to TKI therapy (pre-treatment) as well as following progression on TKI therapy.

Test Requirements
Formalin Fixed Paraffin Embedded (FFPE)Tumour specimen: A minimum of 20% tumour content is required.

Results Reporting
OncoPanel reports include a list of variants classified into tiers of clinical significance:
TIER I – VARIANTS OF STRONG CLINICAL SIGNIFICANCE
TIER II – VARIANTS OF POTENTIAL CLINICAL SIGNIFICANCE
TIER IIIA –VARIANTS OF UNCERTAIN CLINICAL SIGNIFICANCE
TIER IIIB – VARIANTS OF UNCERTAIN FUNCTION
Variants suspected to be of germline origin will be identified on the report as Potential Germline Findings.

Genes Targeted
Either the coding exonic sequence and at least 2 bp of flanking intronic sequence, or known hotspots of clinical importance, are assessed in the following genes.

Reporting of variants in these genes is limited to Tiers I, II, IIIA and IIIB:
AKT1, ALK, ATM, BRAF, BRCA1, BRCA2, CDK4 (codon 24), CDKN2A, EGFR (Exons 18-21), ERBB2, ERBB3, GNA11, GNAQ, HRAS, IDH1, IDH2, KIT, KRAS, MAP2K1, MET

(incl. Intron 13), NRAS, PALB2, PDGFRA, PIK3CA, RET, ROS1, SDHA (excl. exon 14), SDHB, SDHC, SDHD. (Reporting of Tier IIIB variants in the SDHx genes is routinely limited to GISTs, in BRCAx to Ovarian Carcinomas, in CDKN2a and GNAx to Melanomas.)

Reporting of variants in these genes is limited to Tiers I, II and IIIA:
APC, AXIN2, BMPR1A, CDH1, CHEK2, CIC, FUBP1, HOXB13, MLH1, MSH2, MSH6, MUTYH, NF1, NTHL1, PMS2, POLD1 (Codons 473-479 only), POLE (Codons 421-427 only), PTEN, RAD50, RAD51C, RAD51D, SMAD4, STK11, and TP53.

Method
Genomic DNA is extracted from the submitted specimen through bead capture (Promega Maxwell). Genomic DNA undergoes FFPE repair, ligation-based library construction, PCR amplification, hybridization capture, and Illumina sequencing. Single-strand consensus sequences are generated from UMI-indexed reads using fgbio and aligned to the GRCh37 human genome reference using BWA. Variant calling is performed using samtools and VarScan2. Annotation and filtering of variants is performed with Agilent's Alissa Interpret platform. Large copy number alterations (duplication or deletion) > 30 bp, other structural rearrangements including gene fusions, and variants in the promoter and other non-coding regions may not be detected by this test. The sensitivity of the present assay is deemed to be approximately 5% (site-specific analyses) to 10% (mutation screening). Variants present in less than this proportion of assayed alleles are not reported. Variants are interpreted in the context of available clinical information and tiered according to their known or predicted clinical significance (adapted from Li et al. Standards and Guidelines for the Interpretation and Reporting of Sequence variants in Cancer J Mol Diagn (2017) 19:4-23 PMID:27993330). Variants unlikely to be of clinical importance (Tier IV) are not reported.
